# Developing a hybrid technique for energy demand forecasting based on optimized improved SVM by the boosted multi-verse optimizer: Investigation on affecting factors

**DOI:** 10.1016/j.heliyon.2024.e28717

**Published:** 2024-03-26

**Authors:** Anzhong Huang, Qiuxiang Bi, Luote Dai, Hasan Hosseinzadeh

**Affiliations:** a School of Accounting and Finance, Anhui xinhua University, Hefei, 230088, Anhui , China; b School of Management, Guangzhou Xinhua University, Dongguan, 523133, Guangdong, China; c School of Digital Economy and Trade, Wenzhou polytechnic, Wenzhou, 325035, Zhejiang, China; d Shahid Beheshti University, Tehran, Iran; e College of Technical Engineering, The Islamic University, Najaf, Iraq

**Keywords:** *Electricity demand*, *Hybrid optimization technique*, *Support vector machine (SVM)*, *Boosted multi-verse optimizer (BMVO)*

## Abstract

Electricity demand prediction accuracy is crucial for operational energy resource management and strategy. In this study, we provide a multi-form model for electricity demand prediction in China that based on incorporating of an upgraded Support Vector Machine (SVM) and a Boosted Multi-Verse Optimizer (BMVO). The suggested model is proposed to address the shortcomings of existing prediction approaches, which frequently fail to internment the complicated nonlinear interactions between demand for electricity and the variables that influence it. The improved SVM algorithm incorporates a modified genetic algorithm based on kernel function for enhancing the stability of the model. The BMVO technique is employed to optimize the combined model's weights and increase its generalization effectiveness. The suggested approach is tested by real-world Chinese energy demand data. The findings show that it outperforms existing prediction approaches in terms of reliability and precision. Further, the number of samples chosen affects how well the proposed BMVO linked with the Incremental SVM (ISVM) predicts outcomes. Particularly, when 1735 samples are chosen, the lowest level of Mean Absolute Percent Error (MAPE) was noted. The Root Mean Square Error (RMSE) and MAPE values under the proposed BMVO/ISVM model are reduced by 53.72% and 55.22%, respectively, compared to the Artificial Neural Network (ANN) approach reported in literature. Finally, the suggested model is capable of accurately predicting the electricity demand in China and has the potential to be applied to other energy-demand prediction applications.

## 1 Introduction

Predicting energy demand is essential for energy suppliers, decision-makers, and consumers alike [1]. Informed choices about energy production, delivery and consumption may benefit beneficiaries from accurate estimates of energy demand [2,3]. Neural networks have become a powerful resource for predicting energy consumption in recent years [4]. An example of a machine learning algorithm that can recognize complex structures in information and predict outcomes is a neural network [5,6]. Regression modeling and time-series evaluation are two statistical techniques that have historically been used in energy demand prediction [7]. These techniques have drawbacks when it comes to identifying the complicated and nonlinear interactions between variables that impact energy consumption, such as climate trends, economic activity, and population shifts [8]. Neural networks have shown the potential to overcome these limitations by allowing the discovery of complex trends in data [9]. Neural networks are composed of layers of connected nodes that train from data by varying the strength of their connections [10]. Neural networks may train non-linear correlations between input parameters and output predictions using this method [11].

For predicting energy consumption, different neural network models have been employed, for example, feed-forward neural networks, Recurrent Neural Networks (RNNs), and Convolutional Neural Networks (CNNs) [12]. The most popular form of neural network for predicting energy consumption is a feed-forward neural network. An input layer, one or more middle layers, and an output layer make up these networks [13]. Another form of neural network that has been applied to anticipating energy consumption is RNNs. Predicting electricity consumption also uses CNNs [14]. In order to extract features that may be utilized to generate forecasts, CNNs employ filters to the input data. For predicting energy consumption, neural networks provide a number of benefits over conventional statistical techniques, including the capacity to recognize gaps in time and understand nonlinear correlations [15]. This is crucial for predicting energy demand since there might be complicated and nonlinear correlations between the input parameters and the electricity demand [16]. Although neural networks may be used to predict energy consumption, there are drawbacks, including the requirement for a lot of data to train networks [17]. Neural networks need a lot of data to train complex relationships, and it is difficult to get large amounts of energy-demand data.

According to the rising demand for electricity and the need to reduce costs and consumption, optimizing neural networks for electricity demand prediction has assumed increasing importance [18]. Effective demand for electricity prediction is now an essential part of managing electricity due to the growth of clean energy sources and the complicated nature of power systems [19]. Neural networks can be optimized for demand for electricity prediction using hyperparameter tuning, regularization, and transfer learning [20]. Hyperparameter tuning involves modifying the network's variables to determine the best settings for the supplied data, while regularization reduces overfitting and increases the network's capacity for generalization. Models that have been trained can enhance the network's efficiency through transfer learning [21].

Metaheuristics are a type of optimization approach that has been widely employed to solve complex problems. Metaheuristics are especially efficient in managing optimization problems that are difficult to resolve with standard approaches, such as gradient-based optimization methods. The optimization of neural networks is one area where metaheuristics have demonstrated great potential [22]. Numerous metaheuristic techniques have been developed to optimize neural networks for demand for electricity prediction. Genetic algorithms, particle swarm optimization, modeled annealing, and ant colony optimization are examples [23]. The field of utilizing artificial intelligence methods for predicting energy demand has been extensively researched. Below are some of the prominent techniques and approaches that have been investigated to develop accurate and efficient energy demand prediction models.

A case study for predicting short-term power consumption using a combination approach utilizing ANFIS and neural networks improved through the DE algorithm is presented by Yang et al. [24]. This research introduces a new combination prediction method using a Back Propagation (BP) neural network, an Adaptive Network-based Fuzzy Inference System (ANFIS), and a variation of the Seasonal Autoregressive Integrated Moving Average (diff-SARIMA). The integrated technique forecasts using all three techniques and it was capable of reducing mistakes and improving accuracy between actual and predicted values. Results investigations indicated that the provided integrated strategy outperformed the other three independent methods in terms of precision [25]. An integrated neural network framework for electricity demand prediction was developed by Kim et al. [26]. Electricity demand prediction is an open challenge for the proper organizing and running of smart grids, clean energy, and energy bidding processes. A combined demand for an electricity prediction system named (c, l)-Long Short-Term Memory (LSTM) + Convolution Neural Network (CNN) was developed to handle this problem. It considers electricity demand as an important factor and adds various types of background information as background values, such as temperature, humidity and season. Simulations with real-world datasets showed that the proposed hybrid approach outperformed the previous techniques in terms of accuracy.

Monthly electricity consumption prediction using neural network models and Fourier series by González-Romera et al. [27]. This study investigated the periodic behavior of monthly electricity consumption in Spain by removing the pattern from the consumption series. A different hybrid technique was proposed, where a Fourier series predicted the periodic behavior and a neural network predicted the trend. Satisfactory results were produced with a MAPE of less than 2%, which enhances the outcomes when simply neural networks or ARIMA are utilized for the same objective.

Azadeh et al. [28] employed neural network-based yearly power demand prediction in high-energy-consuming industrial sectors. This research describes an ANN technique for calculating the yearly electricity demand in high energy-consuming industrial sectors. The ANN method used a supervised multi-layer perceptron (MLP) to predict yearly consumption with reduced inaccuracy. Empirical data from high energy-consuming businesses in Iran from 1979 to 2003 were utilized to demonstrate the ANN method's effectiveness. ANOVA were used to assess the ANN prediction to actual data and the conventional regression equation to demonstrate its superiority. This was the initial investigation to provide a method for predicting long-term power usage in high-energy-consuming sectors using on the ANN and ANOVA.

Ntow Jnr and Ziggah [29] developed a hybrid model including feature selection technique, particle swarm optimization, and Backpropagation neural network for forecasting electricity demand data. The originality of the data was preserved after the feature selection as compared to what was in the literature. The comparison stage involves seven other models which include hybrid and standalone models. Further, the general stability of the proposed model throughout the training, testing, and validation stages were established. Liu et al. [30] developed a model to predict transportation energy demand employing the ANN based on the Improved Red Fox Optimizer. That model could predict the domestic product growth, population, and vehicles number by 5.5 %, 4.8 %, and 4.2 %, respectively. Aslan and Beşkirli [31] developed an improved arithmetic optimization algorithm method for solving energy demand forecasting problem. That model was based on the basic arithmetic optimization algorithm position update rules. The linear regression model was created by using the Turkey's GDP, population, export and import data for the period 1979–2011 years. The experimental results of the model were compared with state-of-the-art population-based algorithms. Ghadimi et al. [32] reported that the sewing training-based optimization algorithm could be a viable option for energy consumption forecasting.

Ekonomou et al. [33] employed artificial neural networks to anticipate Greek long-term energy consumption. This article uses artificial neural networks (ANN) to forecast Greek long-term energy usage. The multilayer perceptron model (MLP) was employed to choose the one with the highest generalizing capacity. Actual recorded input and output data were utilized in the training, verification, and testing processes. The generated ANN model outperformed linear regression, support vector machine, and real-world energy consumption data in terms of accuracy. This strategy can help with the effective implementation of energy policies since it influences capital investment, sustainability, revenue analysis, market research management, and supply security.

This study's goal is to design and improve the work of Yao et al. [34] to creating a prediction model for energy demand forecasting. We expand the framework they use in that study to forecast energy demand in China, which is important for long-term forecasting. The implementation of a multi-step ahead prediction model for energy demand forecasting, based on Support Vector Machines (SVM) that have been successfully used in related studies [35], constitutes the key contribution of this study effort. We also suggest using a forgetting factor to improve the SVM model's prediction accuracy. The method is then optimized based on a modified metaheuristic, called Boosted Multi-Verse Optimizer to provide better results. We compare the proposed SVMFF model to some other methods from the literature in order to assess the efficacy of this technique. This study intends to offer insightful information on energy demand forecasting in China, which can help to engineers. Therefore, the main contributions of this research are summarized as follows.

• Providing a multi-form model for electricity demand prediction in China based on a hybrid ISVM/BMVO model.
• Considering the research gap to address the shortcomings of existing prediction approaches, which frequently fail to internment the complicated nonlinear interactions between demand for electricity and the variables that influence it.
• Testing the suggested approach by real-world Chinese energy demand data.
• Achieving a model for accurate electricity demand predicting in China that has the potential to be applied to other energy-demand prediction applications.

## 2 Problem statement

### 2.1 Forecasting methodology

**Fig. 1:**
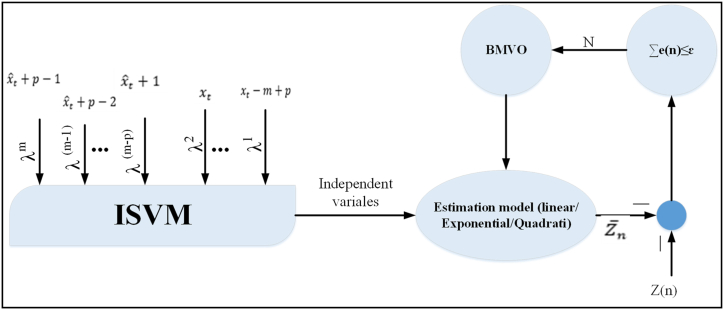
Suggested prediction model in schematic form.

The authors of this study first looked at the correlations between various variables in the fundamental necessary power calculations using an improved support vector machine (ISVM) approach. The objective was to create a reliable forecasting model for China's energy needs. The energy demand forecasting model featured an ideal form based on a newly created modified metaheuristic algorithm, known as Boosted Multi-Verse Optimizer, which is utilized to anticipate the variables. The energy demand forecasting model's parameters are optimized using the boosted multi-verse optimizer, which is based on the suggested ISVM. Fig. (1) shows the optimization-based estimation model using the Boosted Multi-Verse Optimizer.

The model accounts for the different ways in which the energy demand equations can be expressed as well as the variables that impact China's energy demand, including GDP, population, economic structure, urbanization rate, and energy structure. The model's parameters are estimated using the Boosted Multi-Verse Optimizer, and the model is then used to forecast China's energy consumption.

### 2.2 Prediction arrangement

**Fig. 2:**
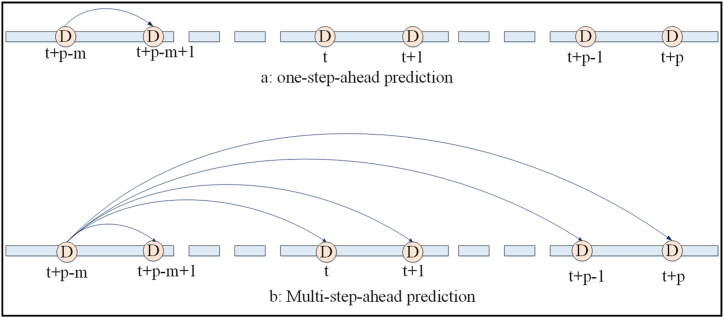
One-step and multi-step forecasting for energy demand.

The characteristics of the multi-step-ahead (MS) energy demand forecasting methods depend not only on the values observed but also on the earlier predictions. Thus, employing general nonlinear input-output models, the recursive relationship between inputs and outputs in MS forecasting can be declared as equation (1):

(1) $$\left\{ \begin{array}{c} {\hat{x}}_{t+p}=F(x_{t+p-m},\ldots ,x_{t},\ldots ,{\hat{x}}_{t+p-2},{\hat{x}}_{t+p-1},p<m\\ {\hat{x}}_{t+p}=F(x_{t+p-m},\ldots ,x_{t+p-2},{\hat{x}}_{t+p-1},p\geq m \end{array}\right.$$

where, m stands for the number of inputs, p describes the steps ahead number for the p-step ahead forecasting model, $x_{t+p-m}$ signifies an observation, ${\hat{x}}_{t+p}$ signifies the output estimation at time t+p, F(.) specifies the MS horizon, The model input comprises all prediction values if p≥m and observation and prediction values if p<m, respectively. Fig. (2) illustrates the one-step and multi-step forecasting for energy demand forecasting with reference to multi-step prediction.

### 2.3 Support vector machine

Support Vector Machines (SVM) is a powerful and widely used machine learning algorithm that is first time introduced by Vapnik et al. [36]. The SVM is a supervised learning method that is commonly used for classification and regression tasks [37]. Given a set of data points, the SVM can estimate the function by equation (2):

(2) $$f\left(X\right)=\sum_{i}w_{i}.x_{i}+b_{i}$$

where, X defines the input datapoint, b describes the bias, w stands for the SVM weight, and by considering the Y as the model output, $x,w\in R^{m}$ and $b,y\in R^{n}$.

equations (3), (4) may be converted to a primal objective function to obtain the estimation of w and b:

(3) $$\min\;J=\frac{1}{2}{\left\|w\right\|}^{2}+C\sum_{j=1}^{s}\left[{\hat{\psi }}_{j}-\psi_{j}\right]$$

Such that:

(4) $$\left\{ \begin{array}{c} y_{j}-\left(\sum_{i}w_{i}.x_{i}+b_{i}\right)\leq \varepsilon +{\hat{\psi }}_{j}\\ \left(\sum_{i}w_{i}.x_{i}+b_{i}\right)-y_{j}\leq \varepsilon +\psi_{j}\\ {\hat{\psi }}_{j},\psi_{j}\geq 0 \end{array}\right.$$

where, the boundary of the prediction and observation values is defined by ε, and C describes a regulation value to provide balance between generalization performance and training error. These two variables are user-set parameters.

To deal with the optimization problem's impractical limitations, two positive slack variables ${\hat{\psi }}_{j},\psi_{j}$, are introduced. The following formula, an optimization issue, may often be evaluated by the Lagrange multipliers $\alpha_{j}$ and $\alpha_{j}^{*}$ [equations (5), (6)]:

(5) $$\max\;J=-\frac{1}{2}\sum_{j,k=1}^{s}\left[\alpha_{j}^{*}-\alpha_{j}\right]\times \left[\alpha_{k}-\alpha_{k}^{*}\right]\times <x_{j},x_{k}>+\sum_{j=1}^{s}\alpha_{j}^{*}\left[y_{j}-\varepsilon \right]-\sum_{j=1}^{s}\alpha_{j}\left[y_{j}+\varepsilon \right]$$

Such that:

(6) $$\left\{ \begin{array}{c} \sum_{j=1}^{s}\alpha_{j}=\sum_{j=1}^{s}\alpha_{j}^{*}\\ C\leq \alpha_{j}\leq 0\\ C\leq \alpha_{j^{*}}\leq 0 \end{array}\right.$$

By assuming the weight equations (7), (8):

(7) $$w=\sum_{j=1}^{s}\left(\alpha_{j}-\alpha_{j}^{*}\right)x_{j}$$

We have:

(8) $$f\left(X\right)=\sum_{j=1}^{s}\left(\alpha_{j}-\alpha_{j}^{*}\right)\times <x_{j},x_{k}>+b$$

By considering the kernel function, $K\left(x_{j},x_{k}\right)$, Eq. (8) can be rewritten as follows [equation (9)]:

(9) $$f\left(X\right)=\sum_{j=1}^{s}\left(\alpha_{j}-\alpha_{j}^{*}\right)\times K\left(x_{j},x_{k}\right)+b$$

where, $K\left(x_{j},x_{k}\right)$ describes the kernel function that has been shown to ease mapping usage. $K\left(x_{j},x_{k}\right)$ equals the inner product of two vectors, space $x_{j}$ and $x_{k}$ in the feature space $\varphi \left(x_{j}\right)$ and $\varphi \left(x_{k}\right)$, that is, $K\left(x_{j},x_{k}\right)=\varphi \left(x_{j}\right).\varphi \left(x_{k}\right)$. All necessary computations can be carried out directly in the input space using kernels, eliminating the need to compute the map, φ(x).

### 2.4 Using of the SVMFF for energy demand forecasting

Predicting energy demand can be challenging due to the varying conditions affecting the demand rate. To address this issue, we propose a technique called Multi-Step (MS) prediction based on Support Vector Machines (SVM). The MS-SVM technique uses m observation data to predict the future displacement, where the m+1 value is predicted based on the m observation data. The predicted output is then used as an input for subsequent predictions, enabling the recursive prediction of future values. In summary, the MS-SVM technique recursively predicts future values based on previous observations. This technique can be briefly presented by the following steps:

A) Apply a one-step-ahead forecasting model based on SVM.
B) The recursive principles of p-step-ahead prediction can be used to predict future values by embedding a one-step-ahead estimator based on SVM.

Due to the fact that demand conditions are complex, energy demand will fluctuate over time. A typical SVM, on the other hand, does not take into account time-varying aspects of the data because it implies data in memory (window) using identical weights. A forgetting factor, called $\lambda^{j}$, has been established to provide exponentially fewer weight on past data, here, ($0<\lambda^{j}\leq 1$, 1<j≤m). In the present investigation, $\lambda^{j}$ is utilized to represent the weights between new and old data, i.e. the weight of previous demands (e.g. m,−1,…). When λ=1, the SVM with the forgetting factor and the SVM without the forgetting factor are the same. When λ=0, just the previous energy usage is utilized to anticipate demand. Other previous demands have no bearing on the present demand.

The purpose of this study is to optimal selection of the model parameters, including C and ε in order to properly forecasting of the unknown data. To do so, here, we designed a modified version of a newly introduced metaheuristic algorithm, called Boosted Multi-Verse Optimizer (BMVO), which is explained in the following section.

## 3 Boosted multi-verse optimizer (BMVO)

### 3.1 Inspiration

World commences through a huge burst that is explained by big bang theory. Based on the big bang theory, it is the whole thing's source in this globe, precisely there was not anything before it. The additional latest and famous theory is Multi-verse theory that are well-known among physicists. The main idea of this theory is that there are several big bang and every big bang reasons the world's birth. The phrase multi-verse stays against the world, that denotes to the additional universes' survival, as well as the universe which all creatures are existing in Multiple universes have interaction and may crash together in the multi-verse theory. This theory as well recommends that there may be diverse physical rules in every universe.

The MVO algorithm is inspired by the 3 key ideas the multi-verse theory: wormholes, white holes and black holes. Theoretical models of these 3 significant the multi-verse theory’ constituents: The diverse portions of a world can link with each other in the holes that its name is wormhole. It is in the multi-verse theory performance as space and time travel tunnels place that substances could move promptly among any a world's every corner, even they are able to move from one world to additional world. A white hole is the hole that has disappeared in real world, while in physicists' opinion, the big bang could be noticed by means of a white hole. More importantly, it might be the core constituent aimed at a world's birth. Furthermore, in the multi-verse theory's the cyclic model, big bangs and white holes are produced place that the crashes among parallel world happen. On the other hand, black holes, have been seen regularly, perform totally difference in comparison with the white wholes. They absorb the whole thing comprising light beams by means of their enormously great gravitational force.

Each world has a permanent inflation (inflation ratio) which reasons its development over space. World’ inflation speed is so significant regard to physical rules, black holes, worm-holes, planets, asteroids, developing stars, white holes, and appropriateness aimed at life. Arguably, one of the cyclic multi-verse models that several worlds act together by black, white, and wormholes to touch a steady condition. Reality, the MVO algorithm is inspired by this concepts, that is theoretically and precisely demonstrated in the next subdivision.

### 3.2 MVO algorithm

According to the words that were discussion in the previous segment, a process that is based on the population, splits the examination method to 2 stages: examination as opposed to utilization. MVO is applied the white hole and black hole's perceptions to discover examination spaces. Contrary, the wormholes support MVO in developing the examination spaces. It is supposed that every result is similar to a world and every variable in the result is a thing in that world. Furthermore, it is allocated that every result an inflation rate, which is proportionate to the similar the result's cost function value, and it is utilized that the period time in place of the repetition in this study because it is a joint period in cosmology and multi-verse theory. Throughout optimization, the next regulations are employed to the MVO's world.

- Having the high ratio of inflation, increasing having white hole's chance.
- Having the high ratio of inflation, decreasing having black holes' chance.
- Worlds by high ratio of inflation have tendency to direct substances over white holes.
- Worlds by low ratio of inflation have tendency to obtain a lot of substances over black holes.
- The substances in totally worlds might encounter accidental motion towards the greatest world through wormholes irrespective of the ratio of inflation.

The substances are let transfer among diverse worlds over white and black hole tunnels. As soon as a black and white tunnel is recognized amid both worlds, the world by high ratio of inflation is assumed to own white hole, while the world by low ration of inflation is supposed to have black holes. The substances are then transported from the white holes of the origin world to the last stop world ‘s black holes. This instrument lets the worlds to simply give-and-take substances. To enhance the total ration of inflation of the worlds, it is supposed that the worlds by high ratios of inflation are extremely possible to own white holes.

Contrary, the worlds by low ratios of inflation own a high possibility of owning black holes. Consequently, the chance of moving substances from a high ratio of inflation's world to a low ratio of inflation's world is high. This situation could insure the enhancement of the middling ratio of inflation the complete worlds in excess of the repetitions.

For designing a mathematical model of the white and black hole tunnels and replace the worlds’ substances, a roulette wheel instrument is utilized. At every single repetition, the worlds are sorted on the basis of their ratios of inflation and selected one of them by means of the roulette wheel to own a white hole. The next stages are prepared to accomplish this [equation (10)].

(10) $$z_{j}^{i}=\left\{ \begin{array}{c} z_{k}^{i}\;m1<NI\left(Uj\right)\\ z_{j}^{i}\;m1\geq NI\left(Uj\right) \end{array}\right.$$

here $z_{j}^{i}$ designates the jth worlds' ith parameter, the jth world is depicted by Uj , NIUj is normalized the ratio of inflation in the j-th world, m1 is a random quantity that is ranged between 0 and 1, and $z_{k}^{i}$ designates kth worlds the ith parameter choose by means of a roulette wheel assortment instrument.

White holes’ choice and willpower are ready by means of the roulette wheel, which is on the basis of the normalized ratio of inflation. The less ratio of inflation, the directing substances; high chance though white and black hole tunnels. It is important to notice that NI must be altered to NI aimed at the extension problems.

The investigation is able to be insured utilizing this instrument because the worlds are necessary to replace substances and encounter sudden fluctuations to discover the pursuit space. By considering the mentioned instrument, the world's preserve replacing substances deprived of disorders. To preserve the worlds' variety and accomplish utilization, every world has wormholes to move its substances over space accidently. Precisely, white points signify moved substances over the wormholes. It is possible that the wormholes accidently alter the worlds' substances devoid of attention to the ratio of inflation. To supply local variations aimed at every world and own high chance to enhance the ratio of inflation utilizing wormholes, it is supposed that wormhole tunnels are continuously recognized amid a world and by far the best world shaped so far. This mechanism is described by the next formula [equation (11)]:

(11) $$z_{j}^{i}=\left\{ \begin{array}{cc} \left\{ \begin{array}{cc} Z_{i}+TSR\times \left(\left({hl}_{i}-{ll}_{i}\right)\times r4+{ll}_{i}\right) & r3<\frac{1}{2}\\ Z_{i}-TSR\times \left(\left({hl}_{i}-{ll}_{i}\right)\times r4+{ll}_{i}\right) & \;r3\geq \frac{1}{2} \end{array}\right. & r2<WEP\;\\ z_{j}^{i}\; & r2\geq WEP \end{array}\right.$$

where Xi designates the j-th parameter of by far the best world shaped so far, TDR is a constant, WEP is an additional constant, ll_i_ demonstrations the minor bound of i-th adjustable, hl_i_ is the higher bound of ith adjustable, zi specifies the ith parameter of jth world, and r2, r3, r4 are accidental quantities between from 0 to 1. The pseudocodes are as next (supposing which hl and ll designate the variables' higher and minor bound).

It may be derived from the pseudocodes and precise formulation that there are 2 key constants here: WEP (wormhole existence probability) and TDR (travelling distance rate). The previous constant is aimed at describing the chance of wormhole's presence in worlds. It is mandatory to upsurge linearly over the repetitions to put emphasis on utilization as the growth of optimization progression. TDR is similarly an issue to express the distance rate (deviation) that a substance can be teleported by a wormhole around the best universe obtained so far. Contrary to WEP, TDR is enlarged completed the repetitions to own extra accurate utilization and local exploration about the by far the best gotten world. The adaptive formulations for 2 constants are defined in the next formula [equations (12), (13)]:

(12) $$WPP=\mathrm{MIN}+b\times \left(\frac{\mathrm{MAX}-\mathrm{MIN}}{B_{\max}}\right)$$

(13) TSR=1−b1tBmax1/t

Here, min is 0.2 in this study, max is 1 in this study, b designates the present repetition, and B displays the max amount of repetitions. Also, t is 6 in this study and it explains the utilization precision above the repetitions. The upper t is the earlier and more precise utilization and local examination. It is worth noting that WEP and TDR are able to suppose as coefficients as well, however the adaptive standards regarded to the consequences of this study is recommended.

### 3.3 Boosted multi-verse optimizer

The Multi-Verse Optimizer (MVO) is a novel optimization technique that has demonstrated impressive results in a variety of uses. However, one of the difficulties with MVO is that it can become trapped in local optima at times, resulting in premature convergence and suboptimal solutions.

This work presents an exploration technique for overcoming the issue of premature convergence in MVO to meet this obstacle. The study focuses on the algorithm's population diversity in particular. MVO creates significant population diversity at first owing to random initialization. However, the difference between the populations decreases during the updating stage, reducing the algorithm's diversity and increasing its probability of it getting stuck in local optima.

To overcome this problem, the suggested approaches strive to keep population variety high during the optimization process. This is accomplished by a variety of strategies, involving the incorporation of additional people into the population, making use of mutation operators to discover new parts of the solution space, and the employment of a self-adaptive strategy to alter algorithm parameters depending on the behavior of the population.

Consider the following formula as diversity of the MVO [equation (14)]:

(14) $$D=\frac{1}{n\times L}\times \sum_{j=1}^{n}\sqrt{{\left(F_{i}-{\bar{F}}_{i}\right)}^{2}}\;$$

where, L, ${\bar{F}}_{i}$, and $F_{i}$ represent, in turn, the longest diagonal line length in the solution space, the mean rate of the $F_{i}$, and the cost value of the *i-th* candidate.

Here, if if $D_{low}$ provides greatest value than D, it has a high diverse population. The new updated location can be regarded in this case by using the mutation method as follows [equation (15)]:

(15) $$z_{j}^{i}=\left\{ \begin{array}{cc} \left\{ \begin{array}{cc} Z_{i}+TSR\times \left(\left({hl}_{i}-{ll}_{i}\right)\times \varphi \times \sigma \times \tau +{ll}_{i}\right) & r3<\frac{1}{2}\\ Z_{i}-TSR\times \left(\left({hl}_{i}-{ll}_{i}\right)\times \varphi \times \sigma \times \tau +{ll}_{i}\right) & \;r3\geq \frac{1}{2} \end{array}\right., & r2<WEP\;\\ z_{j}^{i}\; & r2\geq WEP \end{array}\right.$$

where, σ specifies a stated parameter, $\varphi \geq 10\times D_{low}$, and τ∼N(0,1).

Furthermore, as a self-learning mechanism, the anti-cosine mechanism is used as follows [equation (16)]:

(16) $$\delta =\underline{Z}+\left(1+\bar{Z}-\underline{Z}\right)\times \left(1-\arccos\left(\frac{\left(\frac{-2\times N_{it}}{{It}_{\max}\;}+1\right)}{\pi }\right)\right)\times \alpha$$

where, $N_{it}$ and ${It}_{\max}$ represent, in turn, the iterations quantity and the maximum iterations value, and $Z=\left(\underline{Z},\bar{Z}\right)$, $\underline{Z}$ and $\bar{Z}$ describe, in turn, the lower and the higher values of the population.

### 3.4 Algorithm authentication

In order to evaluate the efficiency of the proposed Boosted Multi-Verse Optimizer (BMVO), a series of benchmark functions were employed to validate its performance. Specifically, the study employed the first 10 benchmark functions from the “CEC-BC-2017 test suite,” which are commonly used to evaluate the performance of optimization algorithms. The decision variable ranges for the benchmark functions were set between −100 and 100 to allow a fair comparison with other algorithms. This allowed for a consistent and uniform assessment of the suggested approach, which could then be compared to other improved and well-known algorithms.

The suggested approach was compared to four different optimization techniques, including Owl Search Algorithm (OSA) [38], Pigeon-inspired Optimization Algorithm (PIO) [39], Lion optimization algorithm (LOA) [40], and the standard Multi-verse optimizer (MVO) [41] to validate its performance. These algorithms were chosen for their efficiency in tackling optimization issues as well as their popularity in the literature. The comparison sought to assess the BMVO's efficiency and identify its strengths and drawbacks in comparison to other cutting-edge algorithms. The parameter combinations for the studied algorithms are shown in Table 1.

**Table 1:** Parameter combinations for the studied algorithms.

Algorithm	Parameter	Value	Algorithm	Parameter	Value
OSA [38]	$T_{dead}$	20	PIO [39]	Number of Pigeons	150
	|P|	15		Space dimension	20
	$Acc_{low}$	0.1		Map and compass factor	0.2
	$Acc_{high}$	0.5		Map and compass operation limit	150
LOA [40]	Number of prides	6		Landmark operation limit	150
	Percent of nomad lions	0.5		Inertia factor (w)	1
	Roaming percent	0.5		Self-confidence factor ($c_{1}$)	1.5
	Mutate probability	0.2		Swarm confidence factor ($c_{2}$)	1.5
	Sex rate	0.9	MVO [41]	$WEP_{\min}$	0.3
	Mating probability	0.5		$WEP_{\max}$	1
	Immigrate rate	0.6		Coefficient(P)	5

Given that of the randomized nature of the initializing in the algorithm, the results of optimization techniques may not always generate a globally optimum solution. Nonetheless, they are able to quickly identify a suboptimal solution that is very close to the optimal one. As a consequence, each function was simulated 30 times. As a consequence, critical metrics such as average value (Avg) and standard deviation (StD) values are simplified. The standard deviation assists in the examination of the findings' volatility, whilst the average value provides the mean results for the 30 runs. Table 2 shows a numerical comparison of Table 3 shows a numerical comparison of the modified squirrel search optimizer to other methods to other methods.

**Table 2:** Numerical comparison of the proposed BMVO to other methods.

Benchmark		BMVO	MVO [41]	OSA [38]	PIO [39]	LOA [40]
F1	Avg	0.00	3.79	4.28	4.74	5.29
	StD	0.00	3.16	3.94	4.26	4.96
F2	Avg	0.26	3.52	4.28	4.46	5.08
	StD	0.06	3.34	4.19	4.41	4.93
F3	Avg	0.00	5.76e-5	7.32e-4	6.58e-3	2.52e-3
	StD	0.00	0.00	5.84e-5	4.96e-4	1.97e-4
F4	Avg	0.00	0.00	5.25e-5	7.49e-5	4.63e-4
	StD	0.00	0.00	7.38e-6	9.73e-6	6.82e-5
F5	Avg	0.00	1.93	2.18	2.46	2.83
	StD	0.00	1.79	2.04	2.12	2.51
F6	Avg	0.05	1.69	1.86	2.52	3.72
	StD	0.00	1.43	1.57	2.21	3.48
F7	Avg	0.00	2.72	2.89	3.15	3.65
	StD	0.32	2.44	2.59	3.06	3.27
F8	Avg	0.00	0.67	0.85	0.97	1.11
	StD	0.00	0.39	0.54	0.78	0.92
F9	Avg	0.00	0.00	1.43	1.51	1.63
	StD	0.00	0.00	1.25	1.38	1.53
F10	Avg	0.00	1.35	1.42	1.56	1.87
	StD	0.00	1.23	1.32	1.41	1.72

**Table 3:** Results of comparisons for different number of samples based on the MAPE.

Selected samples #	Set of features directly	Suggested method
1735	16.28	9.16
2057	14.00	10.16
2498	11.71	9.52
2840	15.67	9.74

The recommended BMVO solved the CEC-BC-2017 test suite with the best level of accuracy, according to the data shown in Table 2. This result implies that the suggested strategy is more effective than the other options taken into account. Additionally, among the methods examined, the proposed method has one of the lowest standard deviations (StD) values, indicating greater dependability in addressing optimization problems over a number of runs. The new method's decreased standard deviation value when compared to older approaches demonstrates its improved reliability in providing reliable findings over several assessments.

## 4 Simulation results

### 4.1 System configuration

Due to the stochastic nature of a metaheuristic algorithm, its outcomes can vary each time the algorithm is run. To ensure consistency, we conducted each experiment 30 times. The table shows that, in addition to the proposed Boosted Multi-Verse Optimizer, several other algorithms, including well-established ones, were used to solve the same experimental suite for comparison purposes. To ensure fairness in the comparison, all algorithms were limited to a maximum of 100 iterations (equivalent to 3000 function evaluations) and were programmed in MATLAB R2017b, 64-bit version, and executed on a Windows 10 64-bit laptop with an AMD A4 3600 processor and 8 GB of main memory.

This study works on China power system transformation as the case study and its impact on the spot markets power market in China [42]. To further expand on this topic, the China power system transformation is a comprehensive reform program aimed at modernizing and optimizing the country's electricity sector. One of the key areas of focus for the China power system transformation has been the spot markets power market. This market is where electricity is traded in real-time, and its efficient operation is critical to ensuring a stable and reliable power supply.

To validate the effectiveness of the China power system transformation, the spot markets power market from 2020 to 2022 was chosen as a long-established recommended model. This model likely served as a benchmark for evaluating the impact of the transformation on the market.

### 4.2 Analysis indexes

It's critical to evaluate a forecast model's precision and potency as a predictor of future values. The Mean Absolute Percent Error (MAPE) and the RMSE are two regularly used assessment metrics for assessing forecast models. The RMSE serves as a gauge for the discrepancy between expected and observed values. The average squared difference between each anticipated value and its matching actual value is taken into account in its calculation. The RMSE is helpful in determining the size of forecasting mistakes. A forecast model that has a lower RMSE is more precise.

Another metric for assessing a forecast model's accuracy is the MAPE. The average percent difference between the expected and actual values is what it indicates. The absolute value of the difference between each projected value and its associated real value is taken, divided by the actual value, and then the average of these values is used to determine the MAPE. When assessing forecast accuracy in terms of percentage error, the MAPE is helpful.

The efficacy of a forecast model may be assessed from many angles by utilizing both the RMSE and MAPE. The MAPE measures forecast accuracy in terms of percentage error, whereas the RMSE measures prediction accuracy in terms of absolute error. In equations (17), (18), the mathematical modeling of these two measures has been given [43]:

(17) $$RMSE=\sqrt{\frac{1}{m}\sum_{j=1}^{m}{\left|X_{i}-{\hat{X}}_{i}\right|}^{2}}$$

(18) $$MAPE=\frac{1}{m}\sum_{j=1}^{m}\frac{\left|X_{i}-{\hat{X}}_{i}\right|}{X_{i}}$$

where, $X_{i}$ and ${\hat{X}}_{i}$ represent, in turn, the actual and the forecasted values.

### 4.3 Results

The classifying properties provided by this study are based on the suggested ISVM. An enhanced Support Vector Machine (SVM) model for classifying characteristics is shown in Table 3. Based on the Mean Absolute Percent Error (MAPE) statistic, the suggested model is assessed and contrasted with various sample sizes. The number of samples that were chosen was determined using a straightforward Independent Component Analysis (ICA).

In comparison to current models, the suggested ISVM model offers a number of improvements, including increased accuracy and resilience. To evaluate the suggested model's efficacy and pinpoint areas for development, it is crucial to compare it with various sample sizes. The use of MAPE as a performance indicator offers important insights into the model's precision and propensity to forecast future events. The comparison's results, which are shown in Table 3, demonstrate how well the suggested ISVM model categorizes characteristics. In terms of MAPE, the model performs better than other models that are already in use, demonstrating its higher accuracy and ability to accurately forecast future events.

The findings demonstrate that the number of samples chosen affects how well the proposed Boosted Multi-Verse Optimizer (BMVO) linked with the Incremental Support Vector Machine (ISVM) predicts outcomes. Particularly, when 1735 samples are chosen, the lowest level of Mean Absolute Percent Error (MAPE) is noted.

The average percentage difference between the projected values and the actual values is represented by the MAPE, making it a valuable metric to assess the prediction model's accuracy. A lower MAPE means that the prediction model is more accurate. As a result, the finding that the MAPE is at its lowest level when 1735 samples are chosen leads to the conclusion that this is the best number of samples for the proposed BMVO/ISVM model.

**Fig. 3:**
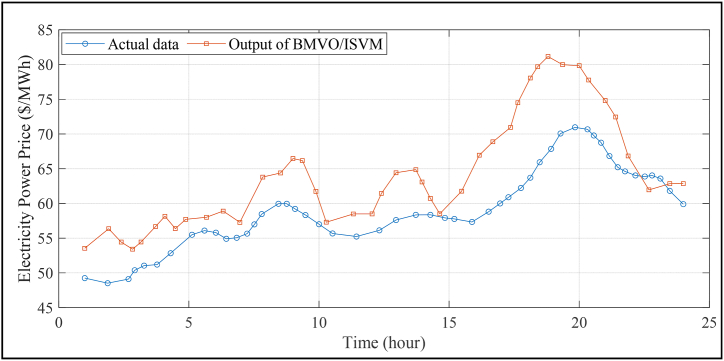
Electricity price prediction results.

When 1735 features are chosen, the best output data has been achieved. This is illustrated in Fig. (3). One of these characteristics is the association between historical pricing over a lengthy period of time; it weakens over time. As a result, it makes sense to delete some qualities. The parameters hour, week, temperature, and season have also been deleted from the list because they have no direct influence on the price of power. As previously stated, ICA is employed here to divide the twenty-four original characteristics into twenty-four major components.

**Fig. 4:**
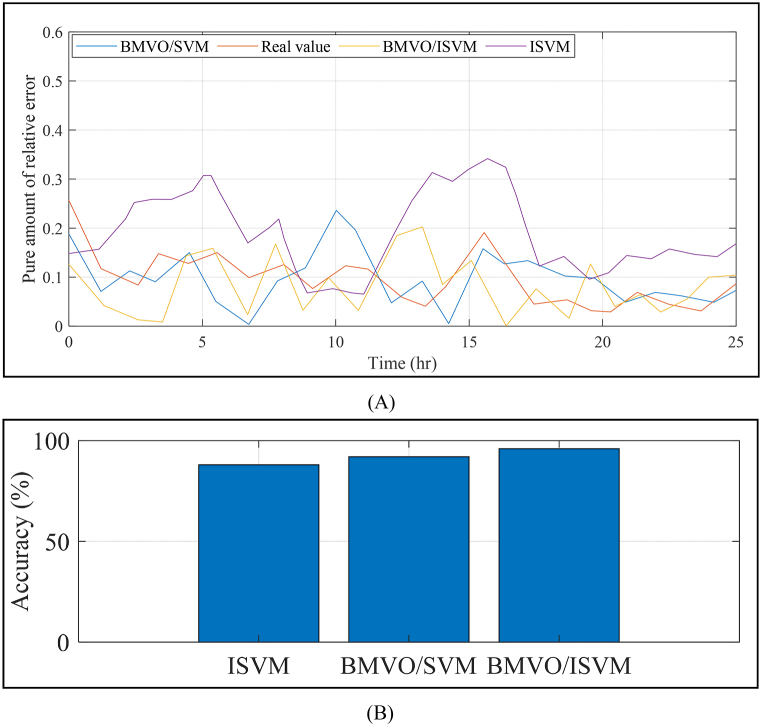
Comparison of data prediction using the suggested method and several existing techniques: Prediction outcomes (A) and accuracy (B).

The suggested technique gives satisfactory support with empirical data, as shown in Fig. (3). The predicted results are still within the acceptable range for the majority of the data. Fig. (4 (A, B)) compares the proposed method to various strategies for data prediction, including ISVM, BMVO/SVM, and BMVO/ISVM.

A comparison is made between the prediction technique's results and those of other published approaches in the literature, such as LSTM neural network [26], Artificial neural network (ANN) [28], Enhanced back-propagation neural network (EBPNN) [44], and CNN-LSTM neural networks [45]. Table 4 shows the forecasting results based on the strategies investigated.

**Table 4:** Forecasting results based on the strategies investigated.

Model	RMSE	MAPE
ANN [28]	8.6	0.201
EBPNN [44]	7.63	0.189
LSTM [26]	5.21	0.130
CNN-LSTM [45]	4.06	0.097
BMVO/ISVM	3.95	0.090

**Fig. 5:**
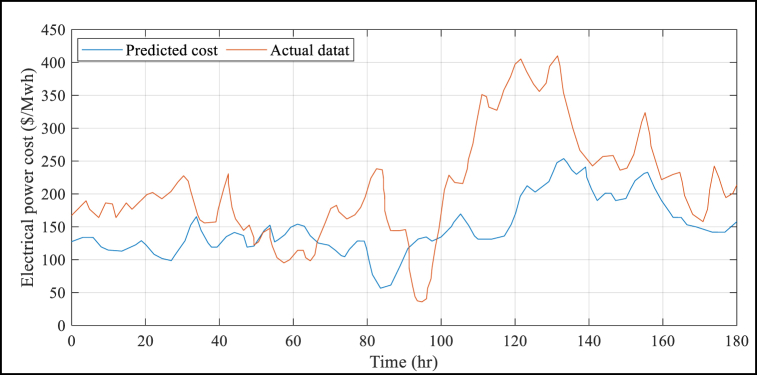
Results of the electrical power cost forecasts from Jan. 1 to Jan. 7.

**Fig. 6:**
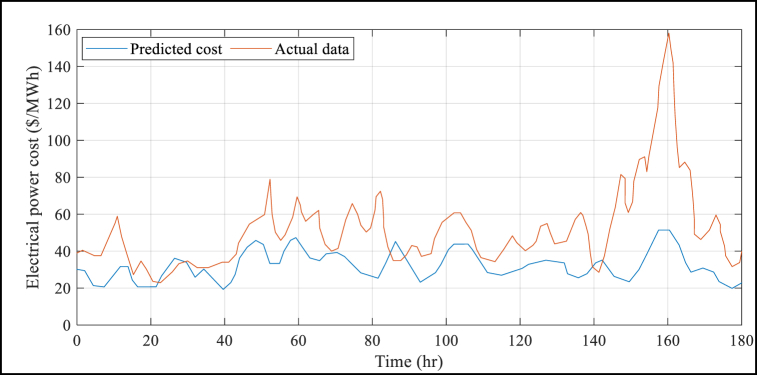
Results of the electrical power cost forecasts from May 30 to June 5.

**Fig. 7:**
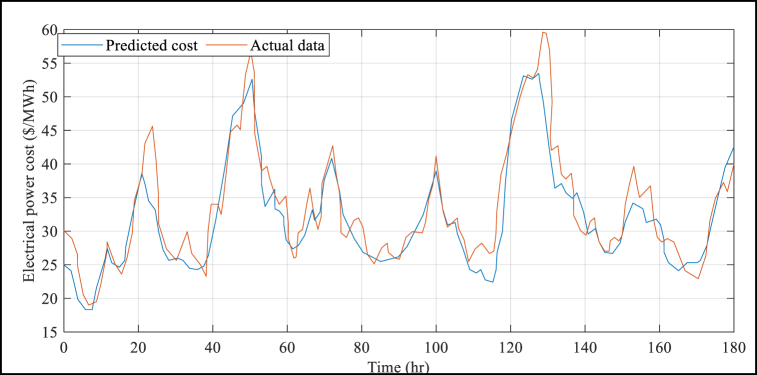
Results of the electrical power cost forecasts from Jul. 9 to Jul. 15.

**Fig. 8:**
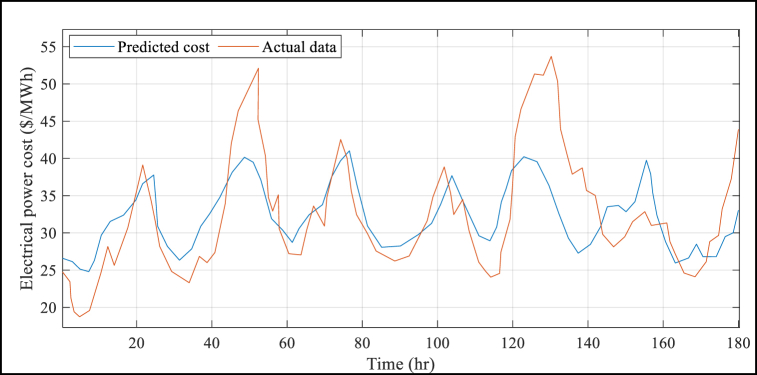
Results of the electrical power cost forecasts from Jul. 10 to Jul. 16.

The suggested BMVO/ISVM method has several advantages, which are further demonstrated by Table 4. This Table shows that the results of the suggested BMVO/ISVM method are superior to those of the other benchmark models. For example, the RMSE and MAPE values under the proposed BMVO/ISVM model are reduced by 53.72% and 55.22%, respectively, compared to the ANN approach reported in literature. Additionally, the study's electricity price forecasts are displayed in Figs. (5) to Fig. (8) for each week of the seasons. These graphs illustrate the weak change laws and large price variations in the sample weeks of electricity prices for each season. Notably, Fig. (5) depicts a significantly higher cost of electricity than Figs. (6) through Fig. (8) (see Fig. 7).

A model's efficiency is frequently best judged by comparing it to other established methodologies. In this example, to further grasp the benefits of the proposed approach, a detailed comparison with alternative methodologies was performed. Table 5 clearly depicts the recommended method's performance, notably in terms of Mean Absolute Percentage Error (MAPE) outcomes when compared to those of other approaches under consideration. It is vital to note that MAPE is a frequently used metric in data analysis and is a measure of forecast accuracy.

**Table 5:** Comparison results of MAPE and RMSE for the suggested strategy against those of other strategies under evaluation.

Method	2nd -8th Jan		May 30-June 5		9th −15th July		9th −15th Oct	
	MAPE (%)	RMSE	MAPE (%)	RMSE	MAPE (%)	RMSE	MAPE (%)	RMSE
BMVO/ISVM	19.26	67.21	21.63	30.24	14.75	7.32	19.35	12.52
ANN [28]	27.54	88.76	31.58	31.12	16.89	9.63	32.54	13.68
EBPNN [44]	24.31	6137	24.37	29.75	17.38	9.69	22.86	13.45
LSTM [26]	25.68	55.97	32.84	28.96	19.84	9.88	28.42	13.58
CNN-LSTM [45]	33.94	95.02	38.92	29.42	21.47	9.89	30.51	15.44

The proposed strategy has a much lower MAPE than alternative options, as demonstrated in Table 5. The MAPE considers both the error-to-true-value ratio and the difference between predicted and actual values. MAPE is a statistician's metric of predicting accuracy. Although RMSE employs Mean error, in which outliers are significant, and because power price oscillation is not stable in winter and spring, and several apex load costs exist, resulting in a significant effect on RMSE, the offered method is by far the most effective in spring and winter.

## 5 Conclusions

In this study, a multi-form framework proposed to forecast energy consumption in China. To increase precision and effectiveness, this approach combined the upgraded Support Vector Machine (SVM) with the Boosted Multi-Verse Optimizer (BMVO). The suggested framework was built to manage a variety of data types, including temporal, geographical, and weather data, all of which have been shown to have a substantial influence on energy consumption. We performed tests utilizing real-world energy consumption information from several areas in China to assess the efficacy of the suggested approach. In terms of precision and effectiveness, the suggested approach beats existing state-of-the-art simulations, including classic SVM and neural network-based methods. Also, we performed sensitivity studies to explore the impact of various input factors on the predicting effectiveness of the suggested approach. The findings show that it outperforms existing prediction approaches in terms of reliability and precision. Further, the number of samples chosen affects how well the proposed BMVO linked with the Incremental SVM (ISVM) predicts outcomes. Particularly, when 1735 samples are chosen, the lowest level of Mean Absolute Percent Error (MAPE) was noted. The Root Mean Square Error (RMSE) and MAPE values under the proposed BMVO/ISVM model are reduced by 53.72% and 55.22%, respectively, compared to the Artificial Neural Network (ANN) approach reported in literature. In general, the suggested multi-form approach has shown to be useful in properly forecasting China's energy consumption. The combined use of enhanced SVM with BMVO has demonstrated tremendous promise in managing numerous and complicated data types, making it a viable strategy for predicting consumption of energy in different locations and nations.

Although the proposed model can achieve better results; however, the use of such optimization algorithms to predict energy demand in power systems is still in the study stages and more detailed studies are needed to achieve an implementable system. It is recommended to analyze more newly developed models and compare the results in future works. In addition, energy demand prediction analysis based on the comparison of energy systems based on fossil power plants with renewable power plants under the proposed model can be an idea for next works.

## Data availability statement

Research data are not shared.

## CRediT authorship contribution statement

**Anzhong Huang:** Formal analysis, Data curation, Conceptualization. **Qiuxiang Bi:** Formal analysis, Data curation, Conceptualization. **Luote Dai:** Formal analysis, Data curation, Conceptualization. **Hasan Hosseinzadeh:** Formal analysis, Data curation, Conceptualization.

## Declaration of competing interest

The authors declare that they have no known competing financial interests or personal relationships that could have appeared to influence the work reported in this paper.

## References

[1] Jain A.K. (2023). 2023 IEEE Power & Energy Society Innovative Smart Grid Technologies Conference (ISGT).

[2] Rodriguez M., Arcos–Aviles D., Martinez W. (2023). Fuzzy logic-based energy management for isolated microgrid using meta-heuristic optimization algorithms. Appl. Energy.

[3] Liu Y. (2023). Model predictive control-based Dual-Mode operation of an energy-Stored Quasi-Z-source Photovoltaic power system. IEEE Trans. Ind. Electron..

[4] Yang C. (2023). Risk-constrained stochastic scheduling for energy hub: Integrating renewables, demand response, and electric vehicles. Energy.

[5] Sun L. (2021). Exergy analysis of a fuel cell power system and optimizing it with Fractional-order Coyote Optimization Algorithm. Energy Rep..

[6] Shirkhani M. (2023). A review on microgrid decentralized energy/voltage control structures and methods. Energy Rep..

[7] Chen L. (2022). Optimal modeling of combined cooling, heating, and power systems using developed African Vulture Optimization: a case study in watersport complex. Energy Sources, Part A Recovery, Util. Environ. Eff..

[8] Yang Z. (2021). Robust multi-objective optimal design of islanded hybrid system with renewable and diesel sources/stationary and mobile energy storage systems. Renew. Sustain. Energy Rev..

[9] Zhang Min (2024). Improved chaos grasshopper optimizer and its application to HRES techno-economic evaluation. Heliyon.

[10] Işık G., Öğüt H., Mutlu M. (2023). Deep learning based electricity demand forecasting to minimize the cost of energy imbalance: a real case application with some fortune 500 companies in Türkiye. Eng. Appl. Artif. Intell..

[11] Zhang J. (2023). Forecast-Assisted Service function Chain Dynamic Deployment for SDN/NFV-enabled Cloud management systems. IEEE Syst. J..

[12] Li Shunlei (2024). Evaluating the efficiency of CCHP systems in Xinjiang Uygur Autonomous Region: an optimal strategy based on improved mother optimization algorithm. Case Stud. Therm. Eng..

[13] Sekhar C., Dahiya R. (2023). Robust framework based on hybrid deep learning approach for short term load forecasting of building electricity demand. Energy.

[14] Hosseini H. (2012). A novel method using imperialist competitive algorithm (ICA) for controlling pitch angle in hybrid wind and PV array energy production system. International Journal on Technical and Physical Problems of Engineering (IJTPE).

[15] Zhu Ligui (2023). Multi-criteria evaluation and optimization of a novel thermodynamic cycle based on a wind farm, Kalina cycle and storage system: an effort to improve efficiency and sustainability. Sustain. Cities Soc..

[16] Pełka P. (2023). Analysis and forecasting of monthly electricity demand time series using pattern-based statistical methods. Energies.

[17] Mehrpooya M. (2021). Numerical investigation of a new combined energy system includes parabolic dish solar collector, Stirling engine and thermoelectric device. Int. J. Energy Res..

[18] Zhang Hua (2024). Efficient design of energy microgrid management system: a promoted Remora optimization algorithm-based approach. Heliyon.

[19] Rezaie M. (2022). Model parameters estimation of the proton exchange membrane fuel cell by a Modified Golden Jackal Optimization. Sustain. Energy Technol. Assessments.

[20] Rao C. (2023). Energy demand forecasting in China: a support vector regression-compositional data second exponential smoothing model. Energy.

[21] Ghadimi Noradin (2023). An innovative technique for optimization and sensitivity analysis of a PV/DG/BESS based on converged Henry gas solubility optimizer: a case study. IET Gener., Transm. Distrib..

[22] Cai W. (2019). Optimal bidding and offering strategies of compressed air energy storage: a hybrid robust-stochastic approach. Renew. Energy.

[23] Han E., Ghadimi N. (2022). Model identification of proton-exchange membrane fuel cells based on a hybrid convolutional neural network and extreme learning machine optimized by improved honey badger algorithm. Sustain. Energy Technol. Assessments.

[24] Yang Y. (2016). Modelling a combined method based on ANFIS and neural network improved by DE algorithm: a case study for short-term electricity demand forecasting. Appl. Soft Comput..

[25] Shen Y. (2023). CEEMD-Fuzzy control energy management of hybrid energy storage systems in electric vehicles. IEEE Trans. Energy Convers..

[26] Kim M. (2019). A hybrid neural network model for power demand forecasting. Energies.

[27] González-Romera E., Jaramillo-Morán M., Carmona-Fernández D. (2008). Monthly electric energy demand forecasting with neural networks and Fourier series. Energy Convers. Manag..

[28] Azadeh A., Ghaderi S., Sohrabkhani S. (2008). Annual electricity consumption forecasting by neural network in high energy consuming industrial sectors. Energy Convers. Manag..

[29] Ofori-Ntow Jnr E., Ziggah Y.Y. (2023). *Electricity demand forecasting based on feature extraction and optimized backpropagation neural network.* e-Prime - Advances in Electrical Engineering. Electronics and Energy.

[30] Liu Y., Yan G., Settanni A. (2023). Forecasting the transportation energy demand with the help of optimization artificial neural network using an improved red fox optimizer (IRFO). Heliyon.

[31] Aslan M., Beşkirli M. (2022). Realization of Turkey's energy demand forecast with the improved arithmetic optimization algorithm. Energy Rep..

[32] Ghadimi N. (2023). SqueezeNet for the forecasting of the energy demand using a combined version of the sewing training-based optimization algorithm. Heliyon.

[33] Ekonomou L. (2010). Greek long-term energy consumption prediction using artificial neural networks. Energy.

[34] Yao J. (2012). Hybrid model for displacement prediction of tunnel surrounding rock. Neural Netw. World.

[35] Li J., Lei Y., Yang S. (2022). Mid-long term load forecasting model based on support vector machine optimized by improved sparrow search algorithm. Energy Rep..

[36] Vapnik V., Guyon I., Hastie T. (1995). Support vector machines. Mach. Learn..

[37] Mou J. (2023). A machine learning approach for energy-efficient intelligent transportation scheduling problem in a real-world Dynamic Circumstances. IEEE Trans. Intell. Transport. Syst..

[38] Jain M. (2018). Owl search algorithm: a novel nature-inspired heuristic paradigm for global optimization. J. Intell. Fuzzy Syst..

[39] Cui Z. (2019). A pigeon-inspired optimization algorithm for many-objective optimization problems. Sci. China Inf. Sci..

[40] Yazdani M., Jolai F. (2016). Lion optimization algorithm (LOA): a nature-inspired metaheuristic algorithm. Journal of computational design and engineering.

[41] Yuan Keke (2023). Optimal parameters estimation of the proton exchange membrane fuel cell stacks using a combined owl search algorithm. Energy Sources, Part A Recovery, Util. Environ. Eff..

[42] Ma X. (2022). Adsorption of low-concentration organic pollutants from typical coal-fired power plants by activated carbon injection. Process Saf. Environ. Protect..

[43] Omidkar A. (2024). Machine learning assisted techno-economic and life cycle assessment of organic solid waste upgrading under natural gas. Appl. Energy.

[44] Zeng Y.-R. (2017). Multifactor-influenced energy consumption forecasting using enhanced back-propagation neural network. Energy.

[45] Kim T.-Y., Cho S.-B. (2019). Predicting residential energy consumption using CNN-LSTM neural networks. Energy.

